# Supplementary material for: Native mass spectrometry combined with enzymatic dissection unravels glycoform heterogeneity of biopharmaceuticals
Source: Nat Commun. 2018 Apr 30;9:1713. doi: 10.1038/s41467-018-04061-7 (PMC5928108; doi:10.1038/s41467-018-04061-7)
Supplement: Supplementary file 1 — Supplementary Information [file 41467_2018_4061_MOESM1_ESM.pdf]

Native mass spectrometry combined with enzymatic dissection unravels glycoform heterogeneity of biopharmaceuticals

Therese Wohlschlager, Kai Scheffler, Ines C. Forstenlehner, Wolfgang Skala, Stefan Senn, Eugen Damoc, Johann Holzmann, Christian G. Huber

SUPPLEMENTARY INFORMATION

1	LPAQVAFTPY	APEPGSTCRL	REYYDQTAQM	CCSKCSPGQH	AKVFCTKTSD	TVCDSCEDST
61	YTQLWNWVPE	CLSCGSRCSS	DQVETQACTR	EQNRICTCRP	GWYCALSKQE	GCRLCAPLRK
121	CRPGFGVARP	GTETSDVVCK	PCAPGTFSNT	TSSTDICRPH	QICNVVAIPG	NASMDAVCTS
181	TSPTRSMAPG	AVHLPQPVST	RSQHTQPTPE	PSTAPSTSFL	LPMGPSPPAE	GSTGDEPKSC
241	DKTHTCPPCP	APELLGGPSV	FLFPPKPKDT	LMISRTPEVT	CVVVDVSHED	PEVKFNWYVD
301	GVEVHNAKTK	PREEQYNSTY	RVVSVLTVLH	QDWLNGKEYK	CKVSNKALPA	PIEKTISKAK
361	GQPREPQVYT	LPPSREEMTK	NQVSLTCLVK	GFYPSDIAVE	WESNGQPENN	YKTTPPVLDS
421	DGSFFLYSKL	TVDKSRWQQG	NVFSCSVMHE	ALHNHYTQKS	LSLSPGK	

Supplementary Figure 1. Amino acid sequence of Etanercept. *N*-glycosylation sites (Asn 149, 171, 317) are indicated in red; *O*-glycosylation sites are shown in blue[1]. The IdeS protease cleaves between the two boxed glycine residues. The C-terminal lysine residue (highlighted in grey) is absent in the most abundant proteoform[2]. Cysteine residues forming intermolecular disulfide bonds are highlighted in yellow. The TNFR domain contains 11 intramolecular disulfide bonds[3], the Fc domain comprises two intramolecular disulfides (not highlighted).

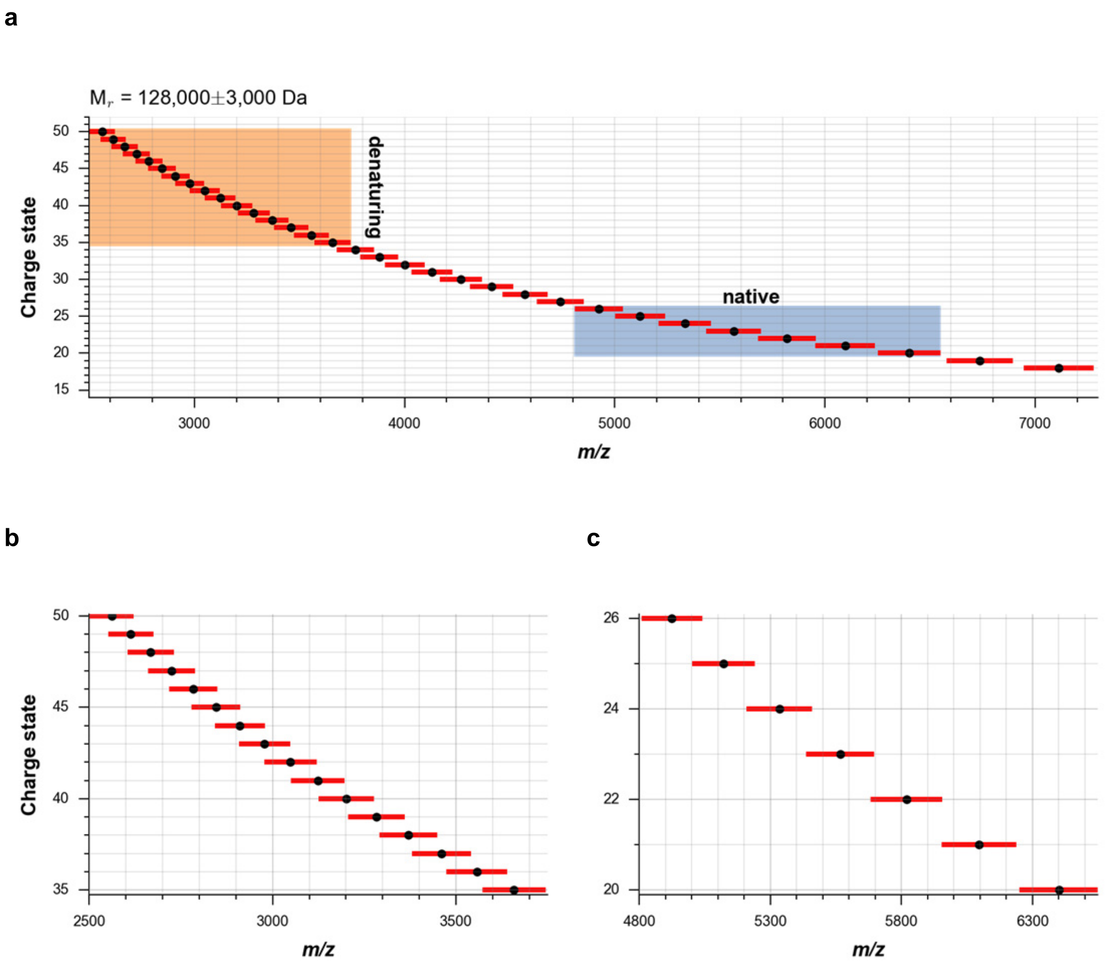

Supplementary Figure 2. Charge state dependent spatial resolution of multiply charged signal clusters under denaturing and native conditions. (**a**) The widths of signal clusters (red bars) arising from a theoretical mass of 128,000 ± 3,000 Da, typical of intact Etanercept including its most abundant glycoform decoration, at different charge states are displayed at corresponding *m/z*. The orange area indicates charge states typically observed under denaturing conditions; the blue area highlights charge states obtained by native MS. Zooms of the plot displaying denaturing and native conditions are shown in (**b** and **c**), respectively.

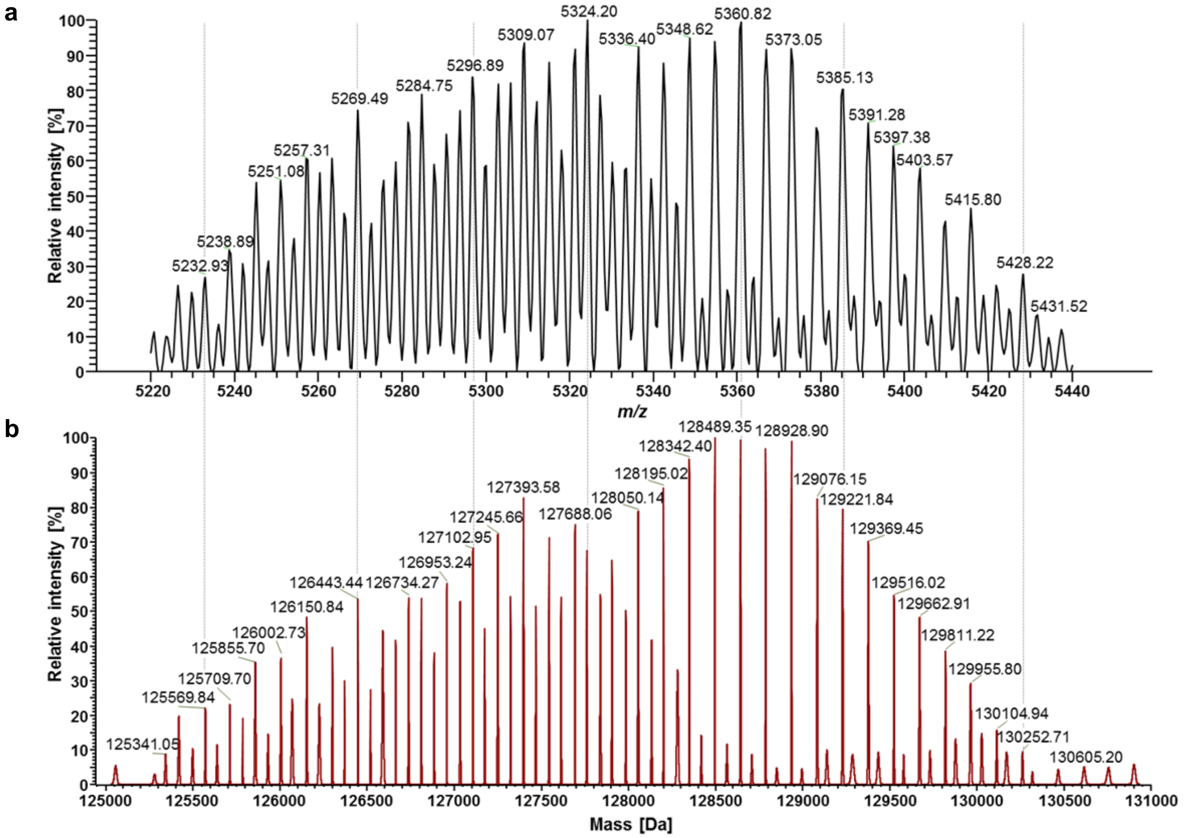

Supplementary Figure 3. (**a**) Zoom into charge state 24+ of intact Etanercept analyzed under native conditions at R_{set} = 17,500 at *m/z* 200 and (**b**) deconvoluted mass spectrum. Dotted lines indicate exemplary signals in the raw spectrum and the corresponding signals in the deconvoluted mass spectrum. Although relative intensities of the different signals are generally consistent between raw and deconvoluted spectrum, large signals tend to be overestimated while small signals are often slightly underestimated in the deconvoluted spectrum. The ReSpect^{TM} algorithm from Positive Probability Ltd. (http://www.positiveprobability.com) employed in this study is part of the Thermo Fisher Scientific BioPharma Finder^{TM} 1.0 SP1 software and relies on probability-based data reconstruction methods that compute the most likely position and intensity of the centroids that are consistent with the data and user models[4]. Notably, peak widths in the deconvoluted spectrum do not arise from natural isotope distribution or instrumental dispersion. Instead, they represent estimated errors in the deconvoluted uncharged centroid masses. The latter are computed from several charge states in the raw spectrum, which increases their probability of accurately predicting the average mass of the corresponding species. Therefore, for computation of glycoform assignments we utilized the average masses obtained by deconvolution. A file containing the observed average masses and their underlying *m/z* values is supplied in Supplementary Data 1.

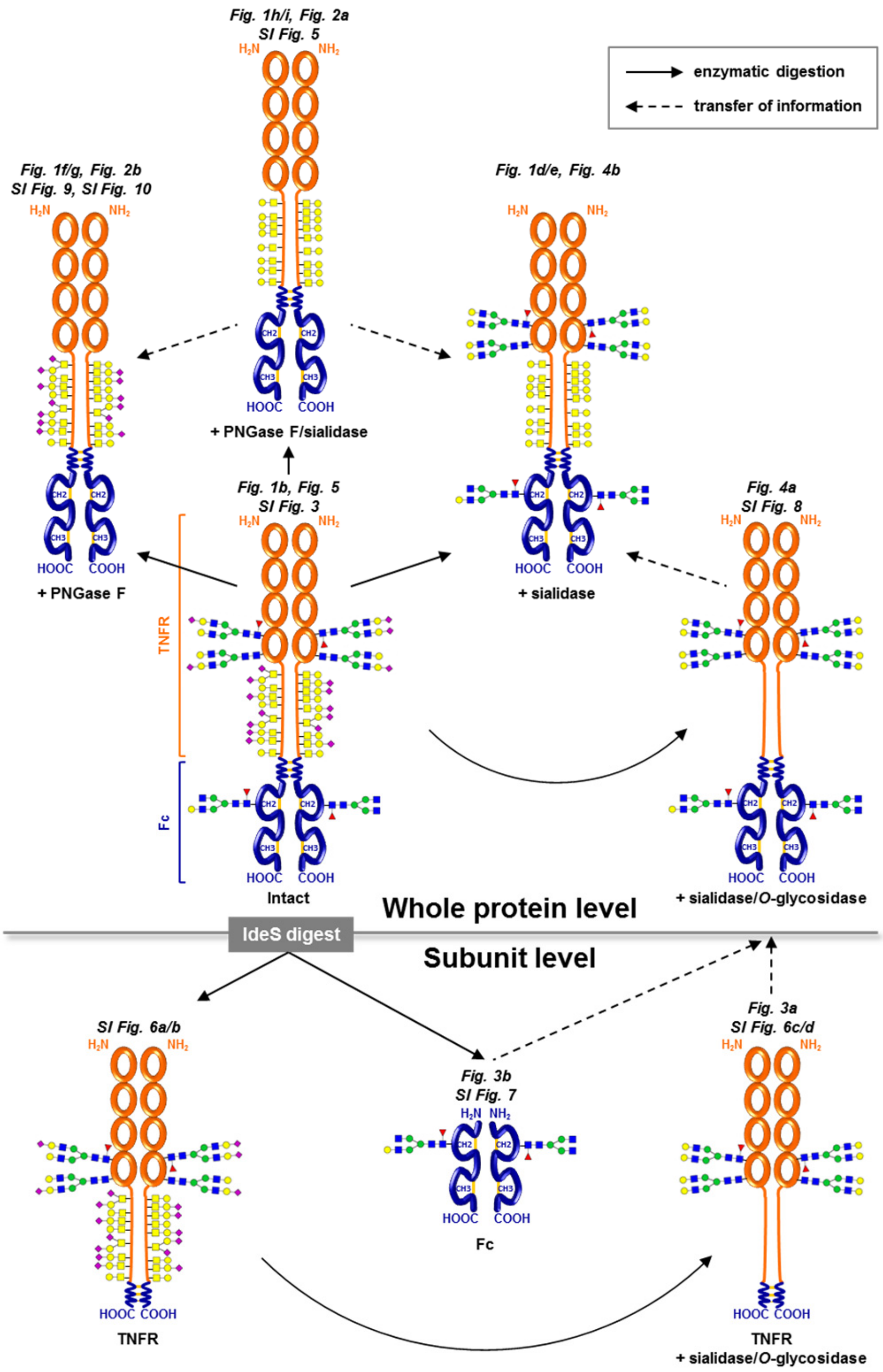

Supplementary Figure 4. Schematic representation of enzymatic digests of Etanercept (full arrows) and transfer of information (dashed arrows). Figure numbers of corresponding mass spectra are indicated above each structure.

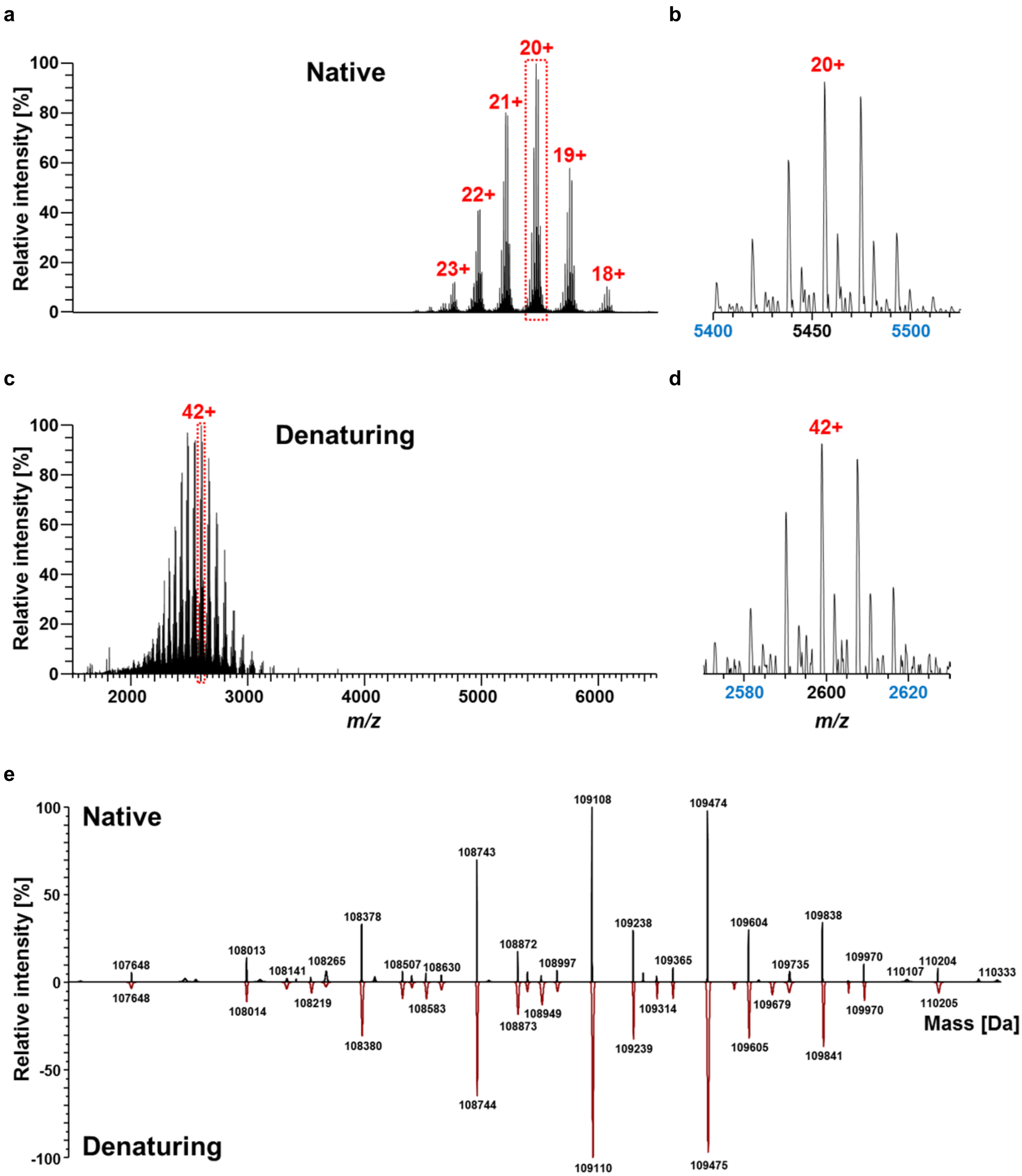

Supplementary Figure 5. Comparison of spectra acquired under native and denaturing conditions, respectively. (**a**) Raw spectra of Etanercept PNGase F/sialidase double digest sprayed from 100 mmol·L^{-1} ammonium acetate (same as in see Fig. 1h and i) or (**c**) 35% acetonitrile/0.5% formic acid, both acquired at R_{set} = 35,000 at *m/z* 200. Charge states are indicated. (**b** and **d**) Zooms into the most abundant charge states are shown (note: *m/z* axis scaling varies). (**e**) Mirror plot of deconvoluted spectra obtained under native and denaturing conditions.

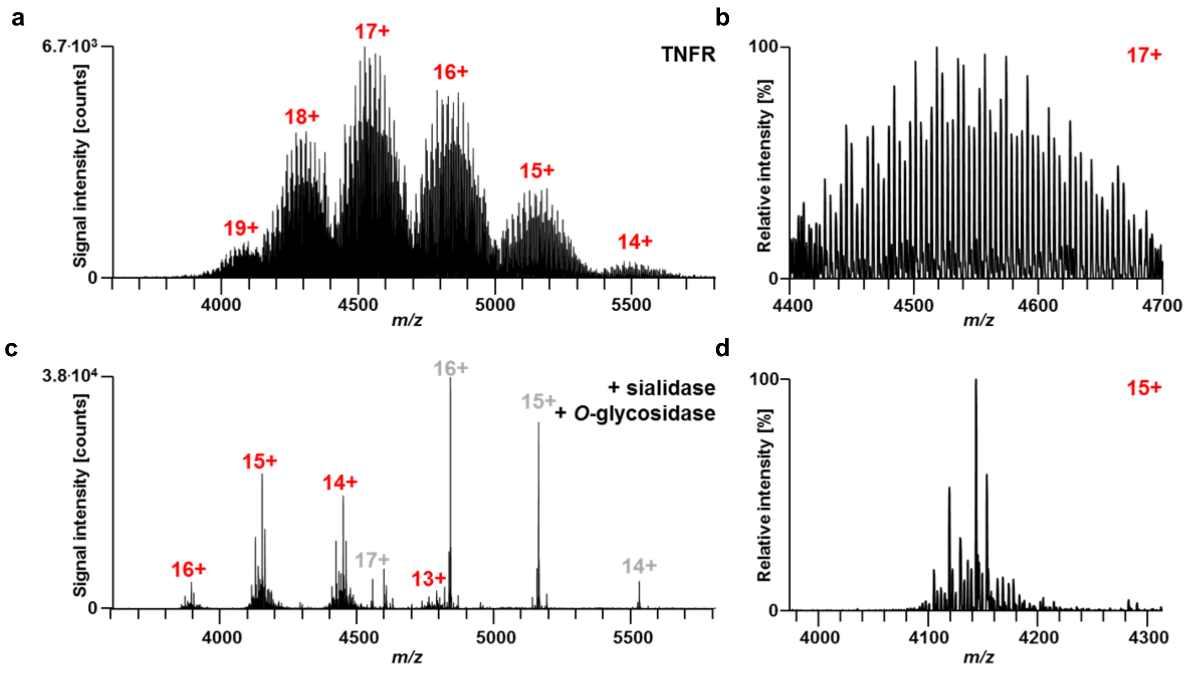

Supplementary Figure 6. Raw spectra of the Etanercept TNFR domain (**a**) before and (**c**) after sialidase/*O*-glycosidase digest. Charge states arising from the TNFR domain are indicated in red. Zooms into the most abundant charge states are shown in (**b** and **d**); x-axis scaling is according to charge state and shows comparable *m/z* range in (b) and (d). Charge states indicated in grey correspond to sialidase present in the sample (**c**). Spectra were acquired at R_{set} = 35,000 at *m/z* 200.

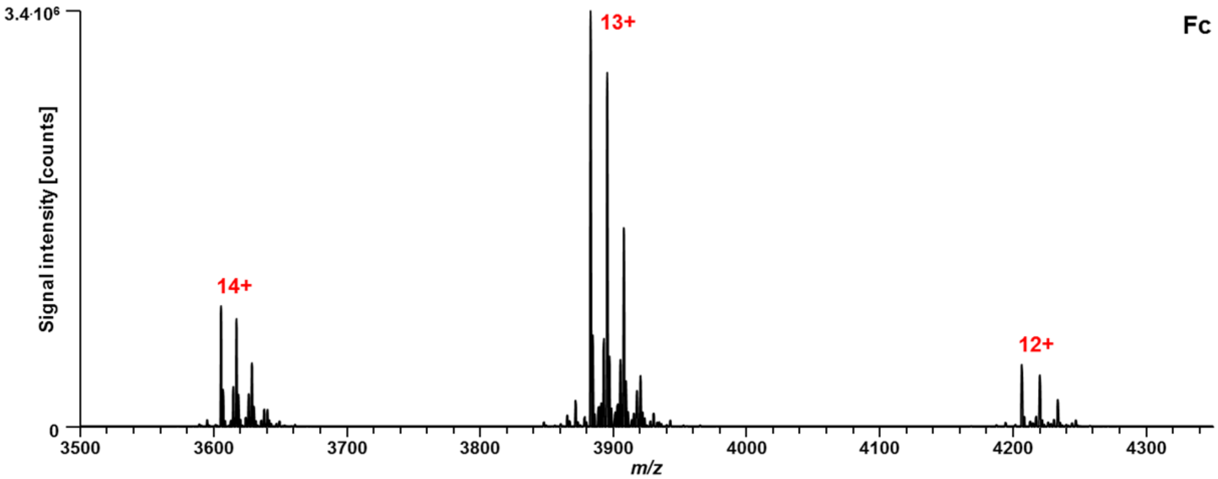

Supplementary Figure 7. Raw spectrum of the Fc domain acquired under native conditions at R_{set} = 35,000 at *m/z* 200. Charge states are indicated. The deconvoluted spectrum is shown in Fig. 3b. The observed masses correspond to dimeric Fc, indicating that native MS enables detection of two non-covalently linked Fc/2 chains, although no intermolecular disulfide bonds are present (Supplementary Fig. 4).

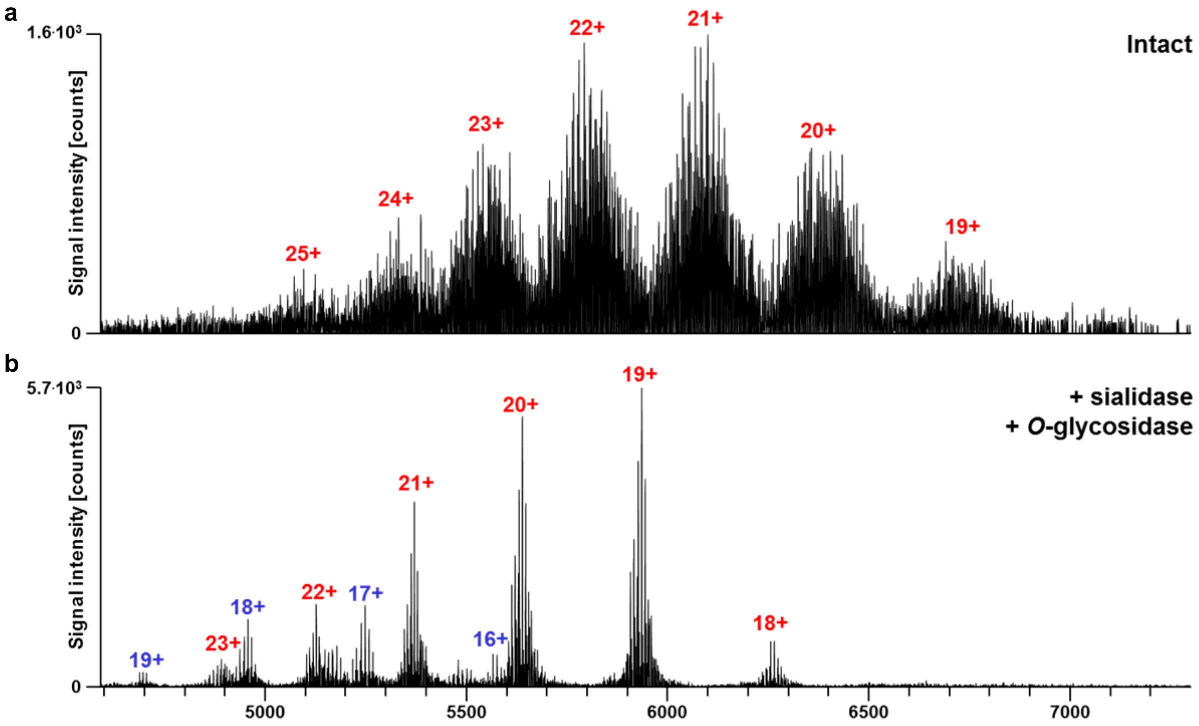

Supplementary Figure 8. Raw spectra of Etanercept (**a**) before and (**b**) after sialidase/*O*-glycosidase digest. Spectra were acquired at R_{set} = 35,000 and R_{set} = 70,000 at *m/z* 200, respectively. Charge states are indicated in red. Charge states labelled in blue in panel b correspond to an Etanercept dimer lacking amino acids 1 to 186 of one TNFR chain.

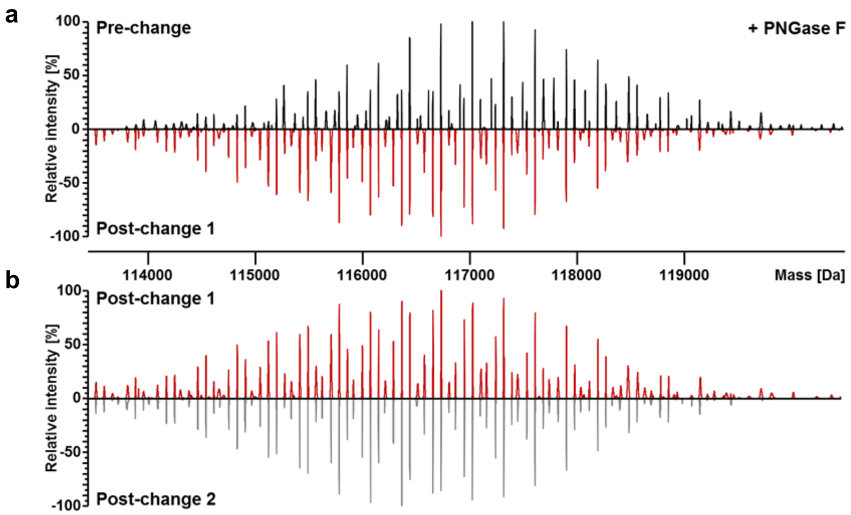

Supplementary Figure 9. Comparison of pre- and post-change batches of Enbrel® by native mass spectrometry upon removal of *N*-glycans. (**a**) Mirror plot of deconvoluted mass spectra of Enbrel® EU pre- and post-change batch 1 after PNGase F digest. (**b**) Mirror plot of deconvoluted mass spectra of Enbrel® EU post-change batches 1 and 2 after PNGase F digest. Spectra were acquired at R_{set} = 35,000 at *m/z* 200.

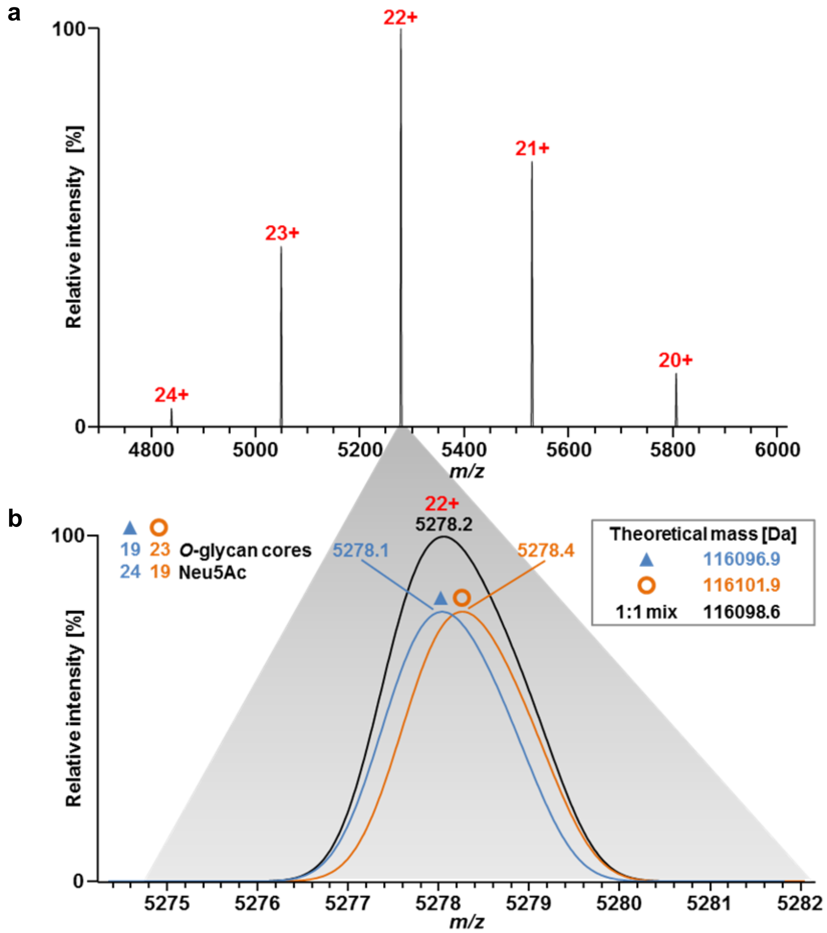

Supplementary Figure 10. Limitations of mass resolution. (**a**) Simulated charge states of the sum of the two possible glycoforms for the most abundant mass of Etanercept PNGase F digest (see Fig. 2b). (**b**) Simulated isotope distributions of the two possible glycoforms (blue and orange line) for the most abundant mass of Etanercept PNGase F digest as well as the sum of a 1:1 mixture of the two variants (black line) at charge state 22+. Theoretical *m/z* values at charge state 22+ and theoretical average masses of each species are shown. Peaks were simulated at an effective resolution of R = 7,100 at *m/z* 5,280 (corresponding to R = 35,000 at *m/z* = 200) using Xcalibur 3.0 software (Thermo Fisher Scientific).

Supplementary Table 1. *N*-glycan structures identified by glycopeptide analysis.

	Residue	Glycan[a)]	Glycan mass [Da][b)]	Fractional abundance [%][c)]
TNFR	N149	A2S1G1	1914.7	41.9%
	N149	A2G2	1623.5	16.4%
	N149	A2S1G1F	2060.9	16.0%
	N149	A2S2	2206.0	9.5%
	N149	A2G2F	1769.6	6.8%
	N149	A2S2F	2352.1	5.9%
	N149	M5	1217.1	3.5%
	N171	A2S1G1F	2060.9	47.3%
	N171	A2G2F	1769.6	21.4%
	N171	A2S2F	2352.1	14.7%
	N171	A2S1G0F	1898.7	6.5%
	N171	A2G1F	1607.5	6.3%
	N171	A2G0F	1445.3	3.8%
Fc	N317	A2G0F	1445.3	47.5%
	N317	A2G1F	1607.5	38.9%
	N317	A2G2F	1769.6	7.0%
	N317	M5	1217.1	4.2%
	N317	A2G0	1299.2	2.4%

Calculated glycan abundances after removal of sialic acids[d)]

TNFR	N149	A2G2	1623.5	67.8%
	N149	A2G2F	1769.6	28.7%
	N149	M5	1217.1	3.5%
	N171	A2G2F	1769.6	83.4%
	N171	A2G1F	1607.5	12.8%
	N171	A2G0F	1445.3	3.8%
Fc	N317	A2G0F	1445.3	47.5%
	N317	A2G1F	1607.5	38.9%
	N317	A2G2F	1769.6	7.0%
	N317	M5	1217.1	4.2%
	N317	A2G0	1299.2	2.4%

[a)]Glycan nomenclature is according to the output from the BioPharma Finder software[5].
[b)]Glycan masses are given as theoretical average masses, in order to enable annotation of masses deduced from native mass spectra obtained for Etanercept.
[c)]Enbrel® EU batch E88057
[d)]Note that upon removal of sialic acids, the abundances of the glycans are summed. For instance, desialylated A2S1G1 (41.9% on N149) and desialylated A2S2 (9.5% on N149) sum up with A2G2 (16.4% on N149) resulting in 67.8% A2G2 on N149.

Supplementary Table 2. Illustration of *N*-glycan structures identified by glycopeptide analysis. In case of possible structural isomers, only one isomer is shown.

**M5**	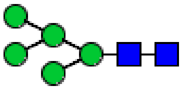	**A2S1G1**	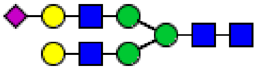
**A2G0**	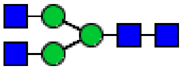	**A2S2**	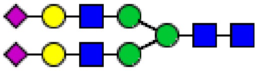
**A2G2**	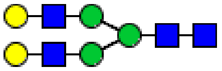	**A2S1G0F**	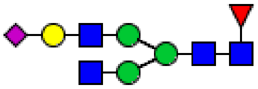
**A2G0F**	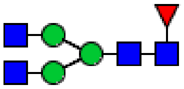	**A2S1G1F**	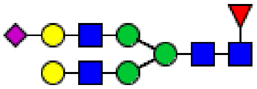
**A2G1F**	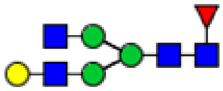	**A2S2F**	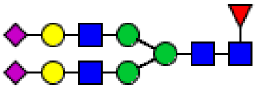
**A2G2F**	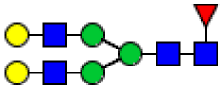		

Supplementary Table 3. Lot numbers of the tested pre- and post-change Enbrel® batches including the labeled expiry dates for human use.

Origin	Lot number	Expiry date	Notes
EU batch	33569	01/2011	pre-change
	E88057	05/2012	post-change 1
	G78712	02/2015	post-change 2
US batch	1042402	05/2016	post-change

Supplementary Data 1. ZIP archive containing raw spectra (in RAW and mzML format); MoFi parameter and output files; processed results and the corresponding processing scripts; glycopeptide data. See the readme file for further details.

SI References

1. Houel S, *et al.* N- and O-glycosylation analysis of etanercept using liquid chromatography and quadrupole time-of-flight mass spectrometry equipped with electron-transfer dissociation functionality. *Anal Chem* **86**, 576-584 (2014).

2. Schiestl M, Stangler T, Torella C, Cepeljnik T, Toll H, Grau R. Acceptable changes in quality attributes of glycosylated biopharmaceuticals. *Nat Biotechnol* **29**, 310-312 (2011).

3. Lamanna WC, *et al.* The structure-function relationship of disulfide bonds in etanercept. *Sci Rep* **7**, 3951 (2017).

4. Ferrige A, Ray S, Alecio R, Ye S, Waddell K. Electrospray-MS Charge Deconvolutions without Compromise – an Enhanced Data Reconstruction Algorithm utilising Variable Peak Modelling. *ASMS conference*, (2003).

5. Zhang Z, Shah B. Prediction of collision-induced dissociation spectra of common N-glycopeptides for glycoform identification. *Anal Chem* **82**, 10194-10202 (2010).
