# Supplementary material for: Native mass spectrometry combined with enzymatic dissection unravels glycoform heterogeneity of biopharmaceuticals
Source: Nat Commun. 2018 Apr 30;9:1713. doi: 10.1038/s41467-018-04061-7 (PMC5928108; doi:10.1038/s41467-018-04061-7)
Supplement: Supplementary file 3 — Description of Additional Supplementary Files [file 41467_2018_4061_MOESM3_ESM.pdf]

Descriptions of Additional Supplementary Files

File Name: Supplementary Data 1
Description: ZIP archive containing raw spectra (in RAW and mzML format); MoFi parameter and output files; processed results and the corresponding processing scripts; glycopeptide data. See the readme file for further details.
